# The Construction and Analysis of the Aberrant lncRNA-miRNA-mRNA Network in Adipose Tissue from Type 2 Diabetes Individuals with Obesity

**DOI:** 10.1155/2020/3980742

**Published:** 2020-04-08

**Authors:** Wei Hu, Yuanlin Ding, Shu Wang, Lin Xu, Haibing Yu

**Affiliations:** ^1^Department of Epidemiology and Medical Statistics, School of Public Health, Guangdong Medical University, Dongguan, Guangdong, China; ^2^Key Laboratory of Prevention and Management of Chronic Kidney Disease of Zhanjiang City, Affiliated Hospital of Guangdong Medical University, Zhanjiang, Guangdong, China

## Abstract

**Background:**

The prevalence of obesity and type 2 diabetes mellitus (T2DM) has become the most serious global public health issue. In recent years, there has been increasing attention to the role of long noncoding RNAs (lncRNAs) in the occurrence and development of obesity and T2DM. The aim of this work was to find new lncRNAs as potential predictive biomarkers or therapeutic targets for obesity and T2DM.

**Methods:**

In this study, we identified significant differentially expressed mRNAs (DEmRNAs) and differentially expressed lncRNAs (DElncRNAs) between adipose tissue of individuals with obesity and T2DM and normal adipose tissue (absolute log_2_FC ≥ 1 and FDR < 0.05). Then, the lncRNA-miRNA interactions predicted by miRcode were further screened with a threshold of MIC > 0.2. Simultaneously, the mRNA-miRNA interactions were explored by miRWalk 2.0. Finally, a ceRNA network consisting of lncRNAs, miRNAs, and mRNAs was established by integrating lncRNA-miRNA interactions and mRNA-miRNA interactions.

**Results:**

Upon comparing adipose tissue from individuals with obesity and T2DM and normal adipose tissues, 364 significant DEmRNAs, including 140 upregulated and 224 downregulated mRNAs, were identified in GSE104674; in addition, 231 significant DEmRNAs, including 146 upregulated and 85 downregulated mRNAs, were identified in GSE133099. GO and KEGG analyses have shown that downregulated DEmRNAs in GSE104674 and GSE133099 were associated with obesity- and T2DM-related biological pathways, such as lipid metabolism, AMPK signaling, and insulin resistance. Furthermore, 28 significant DElncRNAs, including 14 upregulated and 14 downregulated lncRNAs, were found. Based on the predicted lncRNA-miRNA and mRNA-miRNA relationships, we constructed a competitive endogenous RNA (ceRNA) network, including five lncRNAs, ten miRNAs, and 15 mRNAs. KEGG-GSEA analysis revealed that four lncRNAs (FLG-AS1, SNAI3-AS1, AC008147.0, and LINC02015) in the ceRNA network were related to the biological pathways of metabolic diseases.

**Conclusions:**

Through ceRNA network analysis, our study identified four new lncRNAs that may be used as potential biomarkers and therapeutic targets of obesity and T2DM, thus laying a foundation for future clinical studies.

## 1. Introduction

Obesity is a complex multifactorial disease caused by an imbalance between energy intake and consumption. In addition, obesity is a common complication of type 2 diabetes mellitus (T2DM) patients and is significantly associated with an increased risk of T2DM [1–3]. Studies have shown that approximately 80%-90% of T2DM patients can be attributed to overweight or obese [4]. Obese men are seven times more likely to develop T2DM than individuals with a healthy weight range, and obese women have a 12-fold higher risk [5]. In the United States, the prevalence of obesity is as high as 39.6% (41.1% for women and 37.9% for men) [6]. At present, the trend of obesity seems to be stable in most developed countries, but in developing countries, the prevalence of obesity is increasing towards the current level in the United States [7]. The pathophysiological regulatory network of obesity and T2DM has always been a hot spot for scientists and a difficult point of research. An increasing number of studies have suggested that long noncoding RNA (lncRNA) plays an important role in the occurrence and development of obesity and T2DM [8, 9].

lncRNAs are transcripts longer than 200 bases with low protein-coding potential that were originally thought to be by-products of RNA polymerase II transcription and considered “noise” in gene transcription [8, 10–12]. Despite their lack of protein coding capacity, there is increasing evidence that lncRNAs are widely involved in the regulation of gene expression. lncRNAs are generally considered to be important regulators of adipogenesis and adipocyte metabolism [13–17]. lncRNAs also play a role in the pathogenesis of T2DM and T2DM-related complications [18–24]. Competitive endogenous RNA (ceRNA) was first proposed by Salmena et al. [25] and was defined as a class of noncoding RNAs that bind to miRNA. In recent years, an increasing number of reports have showed that lncRNAs can act as ceRNAs by competitively binding to microRNAs (miRNAs), inhibiting miRNA activity and regulating mRNA expression. The lncRNA Gm15290 sponges miR-27b to promote PPAR*γ*-mediated adipogenesis in vitro and to increase fat deposition and body weight in high-fat diet- (HFD-) fed mice [26]. The lncRNA H19 acts as a ceRNA of miR-30a to enhance the expression of the downstream C8orf4, modulating adipogenic differentiation in human adipose tissue-derived mesenchymal stem cells [27]. The lncRNA NEAT1-miR-181b-mLST8 is involved in regulating the mTOR signaling pathway in a T2DM-related ceRNA network [28]. The regulation of the lncRNA H19-miR-140-HE4 axis has a certain effect on hyperglycemia [29]. The lncRNA MEG3 promotes ATF4 expression by competitively inhibiting miR-214, leading to hepatic insulin resistance [30].

The theory of ceRNA has been widely used in the pathogenesis of diseases such as cancer, muscular dystrophy, and neurodegenerative diseases. However, studies on ceRNAs in metabolic diseases, especially obesity and T2DM, are limited. In this study, we identified differentially expressed mRNAs and lncRNAs between adipose tissue of individuals with obesity and T2DM and normal adipose tissues. Then, the lncRNA-miRNA interactions predicted by miRcode were further screened according to MIC > 0.2. Simultaneously, the mRNA-miRNA interactions were explored by miRWalk 2.0. Finally, a ceRNA network consisting of lncRNAs, miRNAs, and mRNAs was established by integrating lncRNA-miRNA interactions and mRNA-miRNA interactions. The aim of this work was to find new lncRNAs as potential predictive biomarkers or therapeutic targets for obesity and T2DM.

## 2. Materials and Methods

### 2.1. Collection of RNA-seq Datasets

The Gene Expression Omnibus (GEO, https://www.ncbi.nlm.nih.gov/geo) database was searched to identify all datasets that have evaluated mRNA, miRNA, and lncRNA expression in adipose tissue samples of T2DM patients with obesity [31]. The following medical subject headings (MeSH) were used for the search: (“diabetes mellitus” OR “type 2, diabetes mellitus” OR “T2DM”) AND “obesity” AND (“adipose tissue” OR “fatty tissue”). The search was restricted to human samples of adipose tissue with a minimum of 3 healthy controls (no T2DM or obesity) and 3 obese T2DM patients. Finally, two RNA sequencing (RNA-seq) datasets, GSE133099 and GSE104674, were obtained. The GSE104674 dataset contains 24 patient samples and 24 healthy control samples based on the GPL16558 AB 5500 Genetic Analyzer (Homo sapiens) platform. The GSE133099 dataset contains 6 patient samples and 6 health control samples based on the GPL16791 Illumina HiSeq 2500 (Homo sapiens) platform (dataset-related information is listed in Table 1).

### 2.2. Identification of Differentially Expressed mRNAs and lncRNAs

The ensemble ID of samples was converted by annoE 1.0.3 (https://github.com/ChrisLou-bioinfo/AnnoENSG2GENE) based on GENCODE 31 (19.06.19) version [32]. The lncRNA, miRNA, and mRNA ensemble IDs that were not included in the GENCODE database were excluded. The edgeR, an R package for differential expression analysis of RNA-seq expression profiles with biological replication, was used to identify significant differentially expressed mRNAs (DEmRNAs) and differentially expressed lncRNAs (DElncRNAs) in samples from patients with T2DM and obesity and in normal samples [33]. All *q* values used the false discovery rate (FDR) to correct the statistical significance for multiple testing. DEmRNAs and DElncRNAs with absolute log_2_FC ≥ 1 and FDR < 0.05 were considered significant and were visualized through volcano graphs.

### 2.3. Gene Ontology and Kyoto Encyclopedia of Genes and Genomes Analysis of DEmRNAs

clusterProfiler, an R package for comparing biological themes among gene clusters [34], was used for Gene Ontology (GO) and Kyoto Encyclopedia of Genes and Genomes (KEGG) analysis of the significant DEmRNAs. GO was used to describe gene functions in three categories: biological process (BP), cellular component (CC), and molecular function (MF). The GO and KEGG analyses were searched for results at the significance level set at adjusted *P* < 0.05.

### 2.4. lncRNA-miRNA-mRNA Network

The miRcode (http://www.mircode.org/) was used to predict interactions between lncRNAs and miRNAs [35]. Moreover, the association between each probe for the DElncRNA and each available miRNA was assessed by using the maximal information coefficient (MIC) computed by minerva, an R package [36, 37]. The lncRNA-miRNA interactions were screened for the significance level set at MIC > 0.2 [38].

The mRNA-miRNA interactions were predicted by miRWalk 2.0 (http://www.umm.uni-heidelberg.de/apps/zmf/mirwalk/), which incorporates 12 algorithms for prediction (TargetScan, RNAhybrid, RNA22, PITA, PicTar2, miRWalk, Microt4, miRNAMap, miRDB, mirBridge, miRanda, and miRMap) [39]. The target miRNAs identified by at least seven algorithms were selected for further analysis.

Then, a lncRNA-miRNA-mRNA network was established by integrating lncRNA-miRNA interactions and mRNA-miRNA interactions and was visualized with Cytoscape 3.7.1 software [40].

### 2.5. KEGG Analysis for the Target miRNAs

The mirPath v.3 (http://www.microrna.gr/miRPathv3/) was used for KEGG analysis of the target miRNAs [41]. The KEGG analysis results were searched for pathways at the significance level of adjusted *P* < 0.05.

### 2.6. Gene Set Enrichment Analysis

clusterProfiler was used for Gene Set Enrichment Analysis (GSEA) of the lncRNAs in the lncRNA-miRNA-mRNA network [42]. The Spearman correlation coefficient between lncRNAs and mRNAs in the GSE133099 dataset was calculated, and the ranked gene list was generated according to the correlation coefficient value. The KEGG-GSEA results were searched for pathways at the significance level of adjusted *P* < 0.05.

## 3. Results

### 3.1. Identification of Differentially Expressed mRNAs

**Figure 1:**
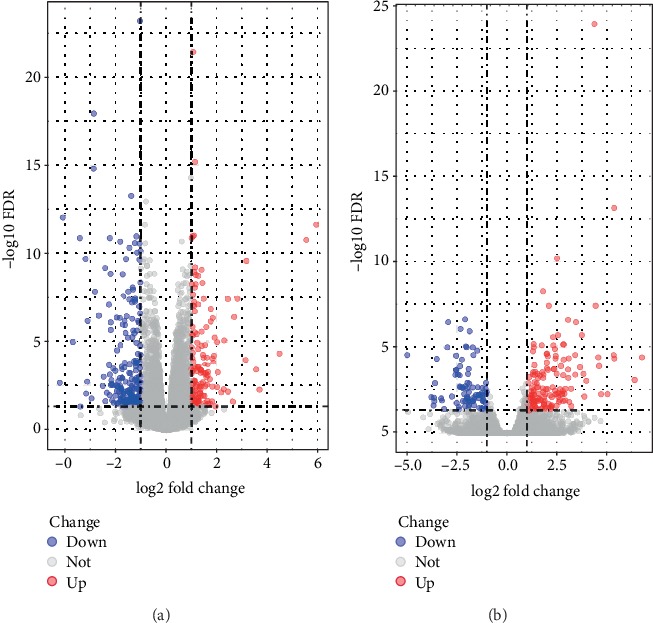
The volcano plot of significant DEmRNAs in GSE104674 (a) and GSE133099 (b). Red dots represent upregulated mRNAs, and blue dots represent downregulated mRNAs.

Two RNA-seq datasets (GSE133099 and GSE104674) were included in our study. A total of 364 significant DEmRNAs, including 140 upregulated and 224 downregulated mRNAs, were found in the GSE104674 dataset (Figure 1(a)); in addition, 231 significant DEmRNAs, including 146 upregulated and 85 downregulated mRNAs, were identified from the GSE133099 dataset (Figure 1(b)).

### 3.2. GO and KEGG Analyses of Significant DEmRNAs

**Figure 2:**
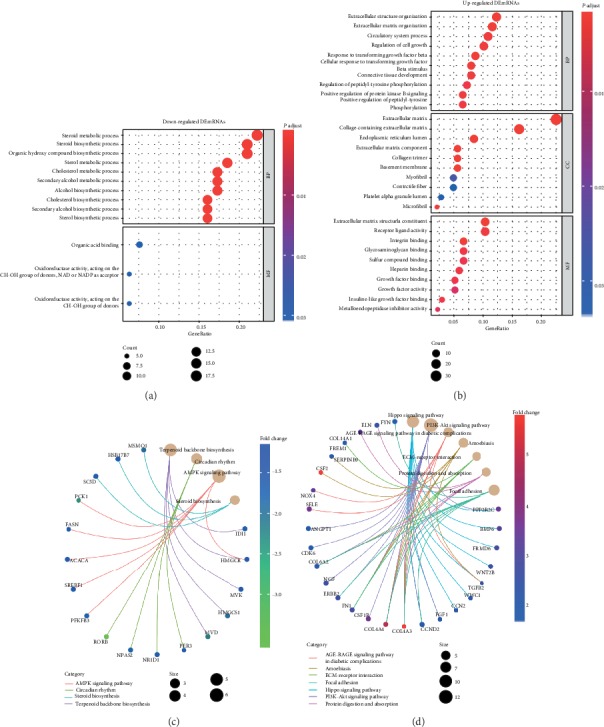
GO and KEGG analyses for significant DEmRNAs in GSE104674. The bubble charts present GO analysis results of downregulated (a) and upregulated mRNAs (b). The network charts present KEGG analysis results of downregulated (c) and upregulated mRNAs (d).

The significantly upregulated and downregulated DEmRNAs of GSE133099 and GSE104674 were utilized for GO and KEGG analyses. For GO analysis of the GSE133099 dataset, when considering BPs, the top three enriched terms of the downregulated DEmRNAs were the steroid metabolic process, steroid biosynthetic process, and organic hydroxy compound biosynthetic process; the top three enriched terms of the upregulated DEmRNAs were the extracellular structure organization, extracellular matrix organization, and circulatory system processes. With regard to MF, the downregulated DEmRNAs were enriched in organic acid binding, oxidoreductase activity acting on the CH-OH group of donors NAD and NADP as acceptors, and oxidoreductase activity acting on the CH-OH group of donors; the upregulated DEmRNAs were enriched in extracellular matrix structural constituents, receptor ligand activity, and integrin binding. In terms of CCs, the extracellular matrix, collagen-containing extracellular matrix, and endoplasmic reticulum lumen are the top three enriched terms in upregulated DEmRNAs only (Figures 2(a) and 2(b)). In the KEGG pathway enrichment analysis of the GSE133099 dataset, when considering downregulated DEmRNAs, the terpenoid backbone biosynthesis, circadian rhythm, AMPK signaling pathway, and steroid biosynthesis were enriched. With regard to upregulated DEmRNAs, the Hippo signaling pathway, PI3K-Akt signaling pathway, and AGE-RAGE signaling pathway in diabetic complications were the top three enriched pathways (Figures 2(c) and 2(d)).

**Figure 3:**
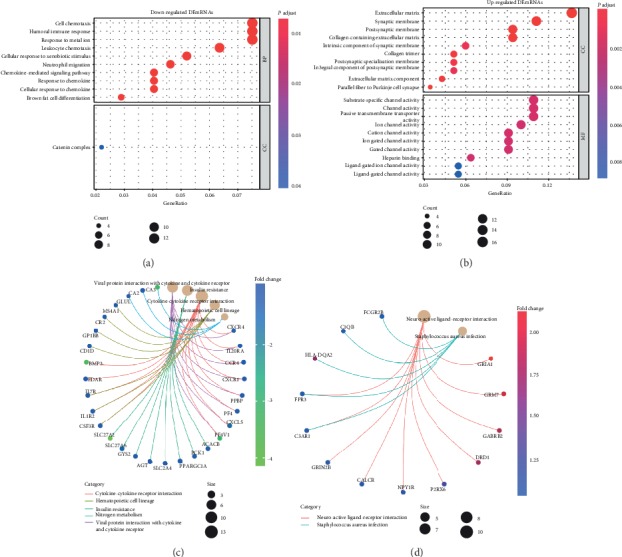
GO and KEGG analyses for significant DEmRNAs in GSE133099. The bubble charts present GO analysis results of downregulated (a) and upregulated mRNAs (b). The network charts present KEGG analysis results of downregulated (c) and upregulated mRNAs (d).

In the GO analysis of the GSE104674 dataset, when considering BPs, the top three enriched terms of the downregulated DEmRNAs were cell chemotaxis, humoral immune response, and response to metalions. With regard to MF, the upregulated DEmRNAs were enriched in substrate-specific channel activity, cation channel activity, and passive transmembrane transporter activity. In terms of CCs, the catenin complex was enriched in downregulated DEmRNAs; the extracellular matrix, synaptic membrane, and postsynaptic membrane were the top three enriched terms in upregulated DEmRNAs (Figures 3(a) and 3(b)). For KEGG pathway enrichment analysis of the GSE104674 dataset, when considering downregulated DEmRNAs, viral protein interactions with cytokine and cytokine receptors, insulin resistance, cytokine-cytokine receptor interactions, hematopoietic cell lineages, and nitrogen metabolism were enriched. With regard to upregulated DEmRNAs, neuroactive ligand-receptor interactions and *Staphylococcus aureus* infection were enriched (Figures 3(c) and 3(d)).

### 3.3. Identification of mRNA-miRNA Interactions

**Figure 4:**
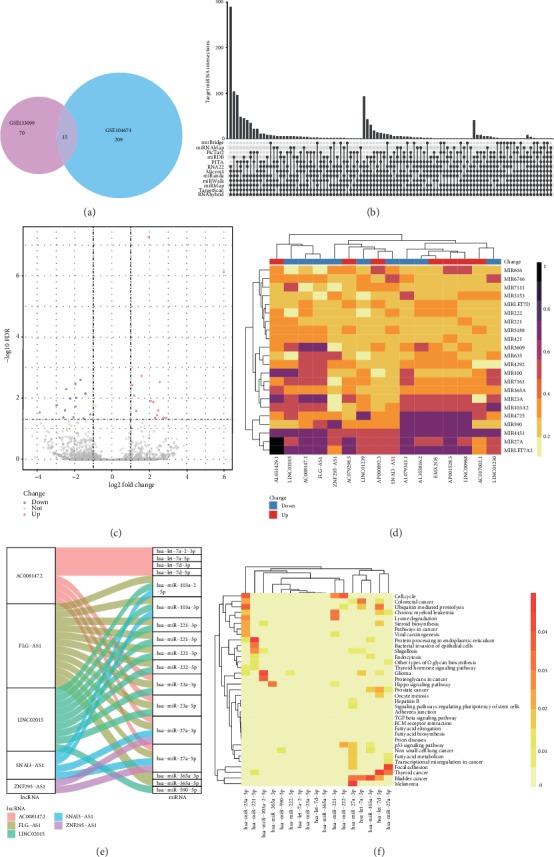
Identification of mRNA-miRNA and lncRNA-miRNA interactions. The downregulation significant DEmRNAs of GSE104674 and GSE133099 intersection was visualized by Venn plot (a). The mRNA-miRNA interactions predicted by miRWalk 2.0 were visualized by UpSet plot (b). The volcano plot of significant DElncRNAs (c). Correlation analysis between lncRNA and miRNA based on MIC (d). The lncRNA-miRNA interactions were visualized by alluvial diagram (e). KEGG pathway analysis for target miRNA (f).

GO and KEGG enrichment analyses showed that significantly downregulated DEmRNAs in the GSE133099 and GSE104674 datasets were involved in obesity- and T2DM-related biological pathways, such as lipid metabolism, AMPK signaling, and insulin resistance. Therefore, the intersection of the significantly downregulated DEmRNAs in the GSE133099 and GSE104674 datasets was selected for further analysis (Figure 4(a)). Subsequently, the 15 selected mRNAs (BMP3, CA3, NDRG4, RORB, LRP1B, NTRK3, RGS2, NPC1L1, CECR2, SYT17, PCK1, SLC27A2, AZGP1, PFKFB3, and ADH1B) were used to predict target miRNAs via miRWalk 2.0. A total of 1178 mRNA-miRNA pairs were found, and these pairs included 720 distinct miRNAs (Figure 4(b)).

### 3.4. Identification and Analysis of lncRNA-miRNA Interactions

The expression profiles of lncRNAs and miRNAs were obtained from the GSE133099 dataset. A total of 28 significant DElncRNAs, including 14 upregulated and 14 downregulated lncRNAs, were found (Figure 4(c) and Table 2).

Thereafter, the DElncRNA-miRNA interactions were identified by MIC correlation testing and the miRcode web tool prediction. Correlations between 16 DElncRNAs and 22 miRNAs were found after applying the MIC threshold (Figure 4(d) and Table 3). Moreover, a total of 438 DElncRNA-miRNA interactions with 18 lncRNAs and 82 miRNAs were found by miRcode. The intersection of the above two groups was selected for further analysis; these intersections contained 35 DElncRNA-miRNA interactions with five lncRNAs and 17 miRNAs (Figure 4(e)).

DIANA-miRPath was exploited to explore the signaling pathways in which the 17 target miRNAs may be involved. These target miRNAs were enriched not only in cancer-related pathways but also in lipid metabolism-related pathways, such as fatty acid biosynthesis, fatty acid metabolism, steroid biosynthesis, and fatty acid elongation (Figure 4(f)).

### 3.5. Construction of the lncRNA-miRNA-mRNA Network

**Figure 5:**
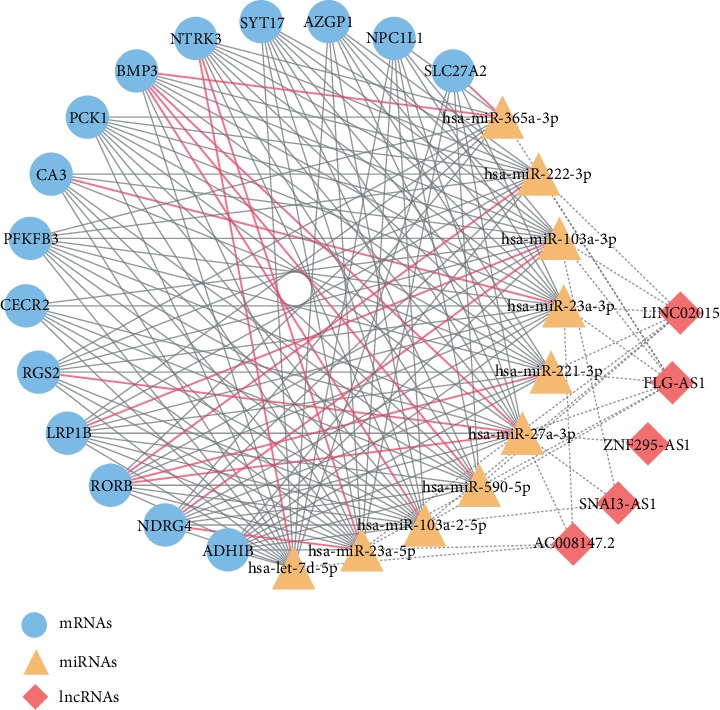
lncRNA-miRNA-mRNA network. This network consisting of five lncRNAs, ten miRNAs, and 15 mRNAs. The lncRNA-miRNA interactions are indicated by dashed lines. The mRNA-miRNA interactions are indicated by solid lines. The solid red line indicates that these interactions are confirmed by at least seven algorithms. The symbols used in the figure are indicated on the right.

When the lncRNA-miRNA pairs and the mRNA-miRNA pairs contained a common miRNA, they were selected for further analysis. After integrating the lncRNA-miRNA interactions and mRNA-miRNA interactions, a lncRNA-miRNA-mRNA network with five lncRNAs (FLG-AS1, SNAI3-AS1, AC008147.2, LINC02015, and ZNF295-AS1), ten miRNAs (hsa-miR-103a-3p, hsa-let-7d-5p, hsa-miR-365a-3p, hsa-miR-222-3p, hsa-miR-590-5p, hsa-miR-103a-2-5p, hsa-miR-23a-3p, hsa-miR-23a-5p, hsa-miR-221a-3p, and hsa-miR-27a-3p), and 15 mRNAs (BMP3, CA3, NDRG4, RORB, LRP1B, NTRK3, RGS2, NPC1L1, CECR2, SYT17, PCK1, SLC27A2, AZGP1, PFKFB3, and ADH1B) was created (Figure 5).

### 3.6. Gene Set Enrichment Analysis

**Figure 6:**
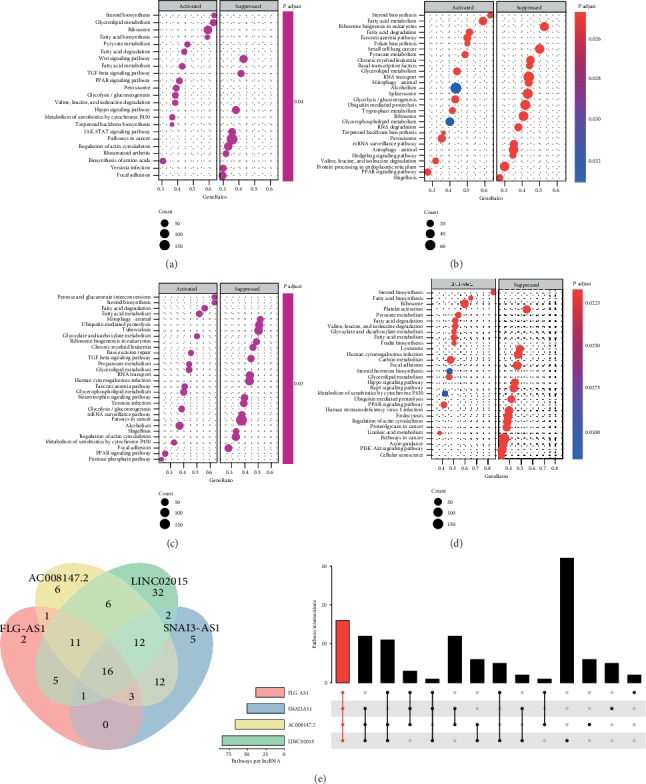
Gene Set Enrichment Analysis for lncRNAs in the ceRNA network. The top 30 pathways of FLG-AS1 (a), SNAI3-AS1 (b), AC008147.0 (c), and LINC02015 (d) enrichment analysis are shown by bubble charts. The Venn and UpSet plots show the pathways for the coenrichment of four lncRNAs (e).

For GSEA analysis, there were 39, 51, 67, 85, and zero enriched pathways for FLG-AS1, SNAI3-AS1, AC008147.0, LINC02015, and ZNF295-AS1, respectively (Figures 6(a)–6(d)). There were 16 intersections of enriched pathways of FLG-AS1, SNAI3-AS1, AC008147.0, and LINC02015, including cellular senescence; ubiquitin-mediated proteolysis; TGF-beta signaling; pyruvate metabolism; fatty acid degradation; peroxisomes; valine, leucine, and isoleucine degradation; steroid biosynthesis; fatty acid metabolism; fatty acid biosynthesis; glycerolipid metabolism; PPAR signaling; carbon metabolism; microRNAs in cancer; Wnt signaling; and glycolysis/gluconeogenesis (Figure 6(e)).

## 4. Discussion

With the development of the social economy, the incidence of obesity and T2DM has increased. It has been proven that the accumulation of adipose tissue, especially abdominal fat, can exacerbate insulin resistance and increase the risk of T2DM [43]. Obesity and T2DM tend to promote the occurrence and development of tumors, which imposes a huge economic burden to the world. In recent years, with the deepening of basic research on obesity and related metabolic diseases, lncRNAs have been identified to have great potential as biomarkers in fat metabolism-related diseases [12]. Recent studies have shown that lncRNAs have clinical application value and are convenient as biomarkers for disease diagnosis [44, 45]. Some lncRNAs have been reported to play a role in obesity or T2DM. However, these studies did not link obesity to T2DM. To understand more about the biological effects of lncRNAs in T2DM patients with obesity, we constructed a ceRNA network in this study, including five lncRNAs, ten miRNAs, and 15 mRNAs.

The KEGG-GSEA results showed that four lncRNAs (FLG-AS1, SNAI3-AS1, AC008147.0, and LINC02015) in the ceRNA network were associated with cancer-related pathways, such as cellular senescence, microRNAs in cancer, and Wnt signaling. Other studies also showed that these lncRNAs were associated with cancers. As shown in the literature, FLG-AS1 may be involved in the pathogenesis of oral cancer [46]; SNAI3-AS1 can promote the growth and metastasis of hepatocellular carcinoma by inducing tumor epithelial to epithelial-mesenchymal transition [47]; LINC02015 is a protective factor for glioblastoma multiforme and is significantly upregulated in metastatic esophageal squamous cell carcinoma [48, 49]. In addition, ZNF295-AS1 is involved in the pathogenesis of epithelial ovarian cancer [50] and lung cancer [51] and can predict survival in patients with gastric cancer [52]. Though they have not been reported in metabolic diseases such as obesity and T2DM, KEGG-GSEA analysis revealed that these lncRNAs were related to the biological pathways of metabolic diseases, such as glucose metabolism-related pathways, lipid metabolism-related pathways, the TGF-beta signaling pathway, and the PPAR signaling pathway. Therefore, the lncRNAs in this network may play an important role in obesity and T2DM. These lncRNAs may act as ceRNAs to regulate other RNA transcripts by competing for shared miRNAs, thus regulating the pathogenesis of obesity and T2DM.

The miRNAs are highly conserved, single-stranded, noncoding small RNAs with a length of 18 to 25 nt [53, 54] that can regulate gene expression by inhibiting the translation of their target mRNAs or reducing their stability at the posttranscriptional level. Studies have shown that one-third of the human genome can be regulated by miRNAs [55], which play an important regulatory role in cancers and metabolic diseases. Most miRNAs in the ceRNA network have been found to be closely related to cancers [56–59]. In addition, Rohm et al. [60] found that the expression of hsa-let-7d-5p was increased in the experimental group compared with the undifferentiated control group during adipogenesis. Lozano-Bartolomé et al. [61] showed that overexpression of miR-23a-3p was involved in insulin signaling in adipocytes in vitro. miR-27a-3p plays an important role in adipogenesis [62], regulation of fat function [63], increase in glycogen storage [64], and regulation of insulin sensitivity [65]. miR-221-3p may affect insulin sensitivity and lipogenesis by regulating ANGPTL8 [66, 67]. miR-222-3p mediates the apoptosis of adipocytes in visceral fat from obese individuals and may attenuate hyperglycemia in a diabetic mouse model [68, 69].

Moreover, most of the mRNAs in the ceRNA network are associated with obesity and T2DM. The decreased expression of ADH1B in adipose tissue was related to obesity, systemic insulin resistance, and a decline in *β* cell function [70], which may be associated with prediabetes [71]. AZGP1 may stimulate lipolysis by regulating the expression of heat-related proteins [72–75]. LRP1B gene polymorphism was associated with insulin resistance, uncontrolled emotional eating, and childhood BMI [76–78]. PCK1 has been proven to be a candidate genetic marker for the risk of diabetes and obesity [79]. PCK1 may also participate in the progression of diabetic neuropathy [80]. PFKFB3 may be a gene that promotes “healthy obesity” [81]. The overexpression of PFKFB3 may lead to an increase in glycolysis [82]. In addition, PFKFB3/iPFK2 is involved in the anti-inflammatory and antidiabetic effects of PPAR*γ* activation [83]. RGS2 may promote adipocyte differentiation and is a key regulator of pancreatic *β* cell survival [84]. SLC27A2 may be a key gene in the PPAR signaling pathway, the adipocytokine signaling pathway, and the insulin resistance pathway [85]. The downregulation of SLC27A2 expression is negatively correlated with diabetes and obesity-related traits, including insulin resistance and BMI [86].

In summary, miRNAs and mRNAs in the ceRNA network were closely related to metabolic diseases such as obesity and T2DM. We have reason to believe that lncRNAs in the ceRNA network can affect the occurrence and development of obesity and T2DM by regulating the activity of target miRNAs and the expression of target mRNAs. However, there is no experimental evidence for the interaction between miRNA-target pairs in the ceRNA network. To improve reliability, these interactions should be verified experimentally. This points the way for our future research. In this paper, we constructed a ceRNA network consisting of lncRNAs, miRNAs, and mRNAs, identifying four obesity- and T2DM-related lncRNAs that have not been reported in metabolic diseases. Therefore, this study may provide new targets for the pathogenesis and treatment of obesity and T2DM.

## Figures and Tables

**Table 1:** Basic information of the datasets from GEO.

Dataset ID	Platform	First author	Year	Region	Sample size (T2DM/N)	Types
GSE133099	GPL16791	Rajan MR	2019	Sweden	6/6	lncRNA, miRNA, mRNA
GSE104674	GPL16558	Stenvers DJ	2019	Netherlands	24/24	mRNA

T2DM: type 2 diabetes mellitus; N: normal.

**Table 2:** The significant DElncRNAs in T2DM with obesity.

lncRNA	logFC	logCPM	*P* value	FDR	Change
AP001432.1	-3.84	-1.85	1.41 × 10^−4^	3.05 × 10^−2^	Down
AC092134.1	-2.94	0.69	6.96 × 10^−5^	1.77 × 10^−2^	Down
ZNF295-AS1	-2.58	1.29	2.32 × 10^−4^	4.35 × 10^−2^	Down
AL353898.3	-2.49	-1.03	1.14 × 10^−4^	2.58 × 10^−2^	Down
LINC01230	-2.44	3.41	1.28 × 10^−5^	5.30 × 10^−3^	Down
Z95114.3	-2.22	-0.34	3.49 × 10^−5^	1.07 × 10^−2^	Down
SNAI3-AS1	-2.04	1.25	2.31 × 10^−4^	4.35 × 10^−2^	Down
LINC02015	-1.93	5.23	7.79 × 10^−6^	3.65 × 10^−3^	Down
AC121247.1	-1.92	-0.15	7.91 × 10^−5^	1.92 × 10^−2^	Down
AL079343.1	-1.89	1.57	3.26 × 10^−5^	1.01 × 10^−2^	Down
AL355816.2	-1.66	0.53	5.35 × 10^−6^	2.69 × 10^−3^	Down
FLG-AS1	-1.51	0.66	2.61 × 10^−4^	4.70 × 10^−2^	Down
LINC01239	-1.41	5.73	2.07 × 10^−5^	7.46 × 10^−3^	Down
AC008147.2	-1.15	3.43	1.68 × 10^−4^	3.46 × 10^−2^	Down
AP001528.3	1.09	4.76	8.79 × 10^−6^	3.98 × 10^−3^	Up
EMX2OS	1.19	3.00	2.55 × 10^−5^	8.61 × 10^−3^	Up
AC017002.1	1.29	3.16	1.21 × 10^−4^	2.70 × 10^−2^	Up
AL031429.1	1.59	5.97	3.36 × 10^−6^	1.97 × 10^−3^	Up
LINC00968	1.96	5.12	7.07 × 10^−12^	5.63 × 10^−8^	Up
AL359091.4	2.07	-0.36	4.27 × 10^−5^	1.27 × 10^−2^	Up
AP000892.3	2.21	2.22	4.83 × 10^−5^	1.37 × 10^−2^	Up
AC025580.2	2.30	-0.46	2.47 × 10^−4^	4.50 × 10^−2^	Up
LINC01173	2.41	-0.04	1.83 × 10^−4^	3.71 × 10^−2^	Up
LINC01503	2.46	-0.02	1.15 × 10^−4^	2.58 × 10^−2^	Up
AC079298.3	2.55	1.98	6.62 × 10^−6^	3.18 × 10^−3^	Up
AC124276.2	2.74	-0.24	2.53 × 10^−4^	4.57 × 10^−2^	Up
LINC01705	2.89	-0.16	2.44 × 10^−4^	4.47 × 10^−2^	Up
AP000879.1	5.95	-0.61	1.71 × 10^−10^	7.94 × 10^−7^	Up

**Table 3:** Basic information of the target miRNA with MIC > 0.2.

Symbol	Genome coordinates	Stem-loop sequence	Mature sequence
MIR616	chr12: 57519163-57519259	hsa-mir-616	hsa-miR-616-5p
			hsa-miR-616-3p
MIR6746	chr11: 61878216-61878278	hsa-mir-6746	hsa-miR-6746-5p
			hsa-miR-6746-3p
MIR7111	chr6: 35470508-35470579	hsa-mir-7111	hsa-miR-7111-5p
			hsa-miR-7111-3p
MIR3153	chr9: 89312225-89312306	hsa-mir-3153	hsa-miR-3153
MIRLET7D	chr9: 94178834-94178920	hsa-let-7d	hsa-let-7d-5p
			hsa-let-7d-3p
MIR222	chrX: 45747015-45747124	hsa-mir-222	hsa-miR-222-5p
			hsa-miR-222-3p
MIR221	chrX: 45746157-45746266	hsa-mir-221	hsa-miR-221-5p
			hsa-miR-221-3p
MIR5188	chr12: 124915547-124915659	hsa-mir-5188	hsa-miR-5188
MIR421	chrX: 74218377-74218461	hsa-mir-421	hsa-miR-421
MIR3609	chr7: 98881650-98881729	hsa-mir-3609	hsa-miR-3609
MIR635	chr17: 68424451-68424548	hsa-mir-635	hsa-miR-635
MIR4292	chr9: 136830957-136831023	hsa-mir-4292	hsa-miR-4292
MIR100	chr11: 122152229-122152308	hsa-mir-100	hsa-miR-100-5p
			hsa-miR-100-3p
MIR7161	chr6: 158609707-158609790	hsa-mir-7161	hsa-miR-7161-5p
			hsa-miR-7161-3p
MIR365A	chr16: 14309285-14309371	hsa-mir-365a	hsa-miR-365a-5p
			hsa-miR-365a-3p
MIR23A	chr19: 13836587-13836659	hsa-mir-23a	hsa-miR-23a-5p
			hsa-miR-23a-3p
MIR103A2	chr20: 3917494-3917571	hsa-mir-103a-2	hsa-miR-103a-2-5p
			hsa-miR-103a-3p
MIR4725	chr17: 31575269-31575358	hsa-mir-4725	hsa-miR-4725-5p
			hsa-miR-4725-3p
MIR590	chr7: 74191198-74191294	hsa-mir-590	hsa-miR-590-5p
			hsa-miR-590-3p
MIR4451	chr4: 85722468-85722533	hsa-mir-4451	hsa-miR-4451
MIR27A	chr19: 13836440-13836517	hsa-mir-27a	hsa-miR-27a-5p
			hsa-miR-27a-3p
MIRLET7A2	chr11: 122146522-122146593	hsa-let-7a-2	hsa-let-7a-5p
			hsa-let-7a-2-3p

## Data Availability

The datasets of differently expressed mRNAs and lncRNAs between adipose tissue from obese T2DM individual and normal adipose tissues were acquired from GEO database, please visit: https://www.ncbi.nlm.nih.gov/geo.
